# Preparation of Phosphorylated *Auricularia cornea* var. Li. Polysaccharide Liposome Gel and Analysis of Its In Vitro Antioxidant Activity

**DOI:** 10.3390/foods13020335

**Published:** 2024-01-20

**Authors:** Wenguang Fan, Xintong Jiang, Qinyang Li, Jiansheng Wang, Minghui Lv, Junmei Liu

**Affiliations:** 1 College of Life Sciences and Engineering, Lanzhou University of Technology, Lanzhou 730050, China; fanwenguang_88@163.com (W.F.); 18088611532@163.com (X.J.); 2 College of Food Science and Engineering, Jilin Agricultural University, Changchun 130118, China; wjs15258622955@163.com (J.W.); colourful2023047@163.com (M.L.); 3 Jilin Province Plant Care Biotechnology Co., Ltd., Changchun 130012, China; 4 College of Chemistry and Chemical Engineering, South China University of Technology, Guangzhou 510641, China; 202367370151@scut.edu.cn

**Keywords:** *Auricularia cornea* var. Li. polysaccharide, phosphorylation, reverse evaporating method, gel, uniform design

## Abstract

In this study, *Auricularia cornea* var. Li. polysaccharides (ACP) were used as the research object to prepare liposome gel and determine its antioxidant activity in vitro. Phosphorylated *Auricularia cornea* var. Li. polysaccharides (P-ACP) were prepared via the phosphorylation of ACP by the phosphate method. Additionally, phosphorylated *Auricularia cornea* var. Li. polysaccharide liposomes (P-ACPL) were prepared using a reverse evaporation method. Finally, phosphorylated *Auricularia cornea* var. Li. polysaccharide liposome gel (P-ACPLG) was prepared by dispersing the P-ACPL in the gel matrix. The results show that the phosphorylation of the P-ACP was 15.51%, the containment rate of the P-ACPL was 84.50%, the average particle size was (192.2 ± 3.3) nm, and the particle size distribution map had a homogeneous peak, resulting in the particle dispersion being uniform and the polydispersion index (PDI) being 0.134 ± 0.021. The average Zeta potential was (−33.4 ± 0.57) mV. In addition, the in vitro antioxidant activity of the P-ACPL was slightly higher than that of the ACP and P-ACP. After the P-ACPL was emulsified into P-ACPLG, the DPPH, hydroxyl radical clearance, and reducing the ability of P-ACPL remained unchanged. In general, the P-ACPLG prepared in this study has good antioxidant activity in vitro and can retain the antioxidant activity of P-ACPL in vitro well.

## 1. Introduction

Edible fungi play a vital role in both nature and the human economy. Polysaccharides are one of the main bioactive components of edible fungi [1,2,3,4]. A large number of studies have found that edible fungi polysaccharides demonstrate anti-oxidation [5], immunomodulatory [6], anti-tumor [7], hypoglycemic [8], anti-aging [9], and other biological activities. ACP is a pure white variant strain with stable genetic traits domesticated and cultivated by the academician Li Yu of Jilin Agricultural University. ACP has good biological effects, including anti-oxidation, anti-inflammation, anti-tumor, liver protection, bacteriostasis, and lowering blood sugar and blood lipids.

The bioactivity of polysaccharides is affected by many factors, and methods are needed to improve their biological activity. Many studies have shown that chemical modification can be used to modify polysaccharides. These modification processes of polysaccharides mainly change the functional groups of polysaccharides by introducing substituents to improve the biological activities of polysaccharides [10], such as anti-oxidation and anti-aging. The chemical modification methods of polysaccharides mainly include phosphorylation, sulfation, acetylation, alkylation, carboxymethylation, etc. [11,12,13], of which phosphorylation is a common modification method for sugars. Phosphorylation is gentler and simpler than other modification methods. Phosphorylated derivatives are usually prepared by phosphorylating reagents, such as phosphorous acid, phosphoric acid, and polyphosphate, etc. For example, Deng et al. [14] reported that the preparation of phosphorylated polysaccharides from *Dictyophora indusiata* were prepared using a phosphoric acid reagent, and it has been found that phosphorylation can enhance the antioxidant activity of polysaccharides in vitro [15,16]. At the same time, in order to effectively improve the bioavailability and biocompatibility of polysaccharides, the material can be prepared at the same time.

Liposomes are a new type of multifunctional self-assembled nanosystem, mainly due to their good biocompatibility, targeting and controllable performance to improve bioavailability and bioactivity, and that they are non-toxic, harmless, and biodegradable, among other advantages [17,18,19]. Due to their unique physiological activity and structural characteristics, liposomes have been widely used as a carrier in medicine, cosmetics, and food [20]. However, the long storage time of liposomes will lead to particle aggregation, poor stability, and other problems.

The combination of liposomes with biopolymers is a promising method to improve the properties of liposomes in vivo and in vitro. Liposomes are usually encapsulated in a variety of polymer substrates, such as gels, films, and nanofibers. These hybrid systems not only preserve the structural integrity and functionality of the encapsulated liposomes but also offer unique benefits with the synergistic efficiency of two different delivery platforms [21,22]. In this study, the gel agent was prepared by dispersing liposomes in the gel matrix. Gels are widely used because they are easy to coat, easy to use, non-toxic, and non-irritating, and they have good biocompatibility and bio-adhesion [23]. In addition, gels can also improve the stability of polysaccharide liposomes and form multi-reservoir systems to enhance slow release and stability. Attempts have been made to solve the problem of poor stability caused by the long-term storage of liposomes [24].

This study aimed to solve the problems of the poor solubility, high molecular weight, and biological activity of ACP and to improve the stability of liposomes and maintain their in vitro antioxidant activity. Therefore, liposomes were dispersed in a gel matrix to prepare a liposome gel. Quality evaluation and in vitro antioxidant activity analysis were performed. The authors of this study preliminarily analyzed the liposome gel to further improve the bioavailability of *Auricularia cornea* var. Li., which can provide a new strategy for the application of *Auricularia cornea* var. Li.in the deep processing field.

## 2. Materials and Methods

### 2.1. Materials

Soybean lecithin and cholesterol were purchased from Shanghai Maclin Biochemical Technology Co., Ltd. (Shanghai, China). Sodium tripolyphosphate, Sodium trimetaphosphate, and glycerol were purchased from Shanghai Yuanye Biotechnology Co., Ltd. (Shanghai, China). *Auricularia cornea* var. Li. fruit-body was developed and provided by academician Li Yu’s team at Jilin Agricultural University. The other chemicals used were analytically pure.

### 2.2. Preparation of Phosphorylated Auricularia cornea var. Li. Polysaccharides

#### 2.2.1. Extraction of *Auricularia cornea* var. Li. Polysaccharides by Water Extract–Alcohol Precipitation

We weighed 3.0 g of *Auricularia cornea* var. Li. powder and dissolved it in distilled water at a liquid/solid ratio of 70:1 until it fully dissolved. After soaking at room temperature for 12 h, the extraction temperature was set at 70 °C and the extraction time was 3.5 h. The extracted solution was centrifuged at 5000× *g* for 30 min, and the supernatant was collected. The supernatant was concentrated to 1/4 of the initial volume using a rotary evaporator at 65 °C, and four times the volume of anhydrous ethanol solution was added to the concentrated solution. After alcohol precipitation at 4 °C for 24 h, the ethanol solution was removed by centrifugation (5000× *g*, 30 min). The obtained polysaccharide was precipitated and fully dissolved with distilled water, concentrated, and the residual ethanol solution was removed. The protein was removed using the Sevag method. After the crude polysaccharide was dissolved, a 1/4 volume of chloroform-n-butanol (4:1, *v*/*v*) mixture was added; the mixture was fully shaken for 20 min then left to stand in the separator hopper. The upper water phase was collected, and the extraction was repeated several times until the protein between the water phase and the organic phase was removed. The polysaccharides of *Auricularia cornea* var. Li. fruit body with the protein removed was obtained by freezing and drying in the upper aqueous phase, which was recorded as ACP.

#### 2.2.2. Preparation of Phosphorylated *Auricularia cornea* var. Li. Polysaccharides

The phosphorylated reagent (70 mg/mL) was prepared by weighing 5.0 g of sodium tripolyphosphate and 2.0 g of sodium trimetaphosphate in distilled water. We added 1.0 g of ACP and 5.0 g of sodium sulfate, adjusted the pH value to 8.6 after dissolution, and reacted the solution at 80 °C for 5 h. At the end of the reaction, 95% ethanol solution was added to increase the volume of the solution to four times that of the original. Then the solution was precipitated for 24 h, centrifuged at 5000 r/min for 10 min to remove the ethanol, and then redissolved. Then, dialysis (8000 Da) was carried out for 48 h, and the solution was concentrated and lyophilized to obtain phosphorylated *Auricularia cornea* var. Li. polysaccharide, which was recorded as P-ACP.

### 2.3. Determination of Phosphate Content

#### 2.3.1. Drawing of Phosphate Standard Curve

The content of phosphate was determined by molybdenum blue colorimetry [25]. First, 0, 0.5, 1.0, 1.5, 2.0, 2.5, 3.0, 3.5, 4.0, 4.5, and 5.0 mL of phosphate standard solution (10 μg/mL of potassium dihydrogen phosphate) was added to the corresponding test tube, and ultra-pure water was added to a volume of 5.0 mL. Then, 3.0 mL of phosphoric acid fixing reagent (a phosphoric acid fixing reagent prepared with 1 mL of 20% ascorbic acid, 1 mL of 3 mol/L sulfuric acid, and 1 mL of 3% ammonium molybdate) was immersed in a water bath at 45 °C for 30 min. After cooling, the light absorption value was measured at 660 nm. The phosphoric acid standard curve was drawn with the phosphoric acid concentration as the horizontal coordinate and absorbance as the vertical coordinate. The corresponding curve regression equation was obtained.

#### 2.3.2. Phosphoric Acid Content Determination

The method for determining the content of phosphoric acid in the sample was as follows: We took a 0.5 g sample and placed it in a beaker, added 1.0 mL of concentrated sulfuric acid and 1.0 mL of concentrated nitric acid successively, and heated it until white smoke was produced. After the solution was cooled, we added 1.0 mL of 30% H_2_O_2_ and heated it again. We repeated the above operation until no white smoke was generated. Then, we added 1.0 mL of 6 mol/L hydrochloric acid and heated it to decompose the residual acid in the solution. Taking 5.0 mL of P-ACP solution, the content of phosphoric acid in the sample was determined by drawing the standard curve.

Phosphoric acid content = (0.2175 A − 0.0075)/S × 100% formula, where A is the absorbance of the sample measured at 660 nm, and S is the mass (g) of the sample.

### 2.4. Preparation and Characterization of Liposomes

#### 2.4.1. Preparation of Liposomes

Cholesterol and soy lecithin with a mass ratio of 5:1 were completely dissolved in 20.0 mL of anhydrous ethanol to form an organic phase by the reverse evaporation–ultrasound method. We slowly added an appropriate amount of P-ACP phosphate buffer solution of 2 mg/mL of aqueous phase at pH = 7.4 (we took 1.36 g of dipotassium hydrogen phosphate, added 0.1 mol/L of 79.0 mL of sodium hydroxide solution, and diluted it to 200 mL with water). The mass ratio of soybean lecithin to P-ACP was 15:1. The mixture was stirred continuously and mixed ultrasonically for 15 min, and then the anhydrous ethanol was evaporated under reduced pressure under a water bath at 45 °C. After the mixture formed a colloidal state, PBS was added to 10.0 mL, and the anhydrous ethanol was evaporated under reduced pressure until completely removed, and then ultrasonic homogenization was performed. After passing through a microporous filtration membrane with a pore size of 0.45 um three times and a microporous filtration membrane with a pore size of 0.22 μm once, the polysaccharide liposome suspension was recorded as P-ACPL and refrigerated under a temperature below 4 °C for preservation. Blank liposomes were prepared in the same way (without adding the P-ACP solution).

#### 2.4.2. Determination of Size Distribution, Zeta Potential and Polydispersion Index of Liposomes

We set the mode to zeta or size. Then, we selected the solvent (water) and set the temperature to room temperature and the equilibrium time to 120 s. Then, we selected the sample tank and selected different modes according to the test zeta potential and size. Then, the scanning parameters were set. Parallel scanning was set for each sample three times; the scanning times were 20 s and the delay time was 3 s. The configured sample concentration was 0.5 mg/mL.

#### 2.4.3. Transmission Electron Microscope Observation

An appropriate amount of the sample was dropped on copper mesh (carbon-coated copper mesh) and then the copper mesh was soaked in 0.01 g·mL^−1^ sodium phosphotungstate solution and stained for 2 min. After removal, the excess solution at the edge was absorbed by filter paper and air-dried. The morphology and structure of the liposomes were observed by transmission electron microscopy.

### 2.5. Preparation of Phosphorylated Auricularia cornea var. Li. Polysaccharides Liposome Gel

We took an appropriate amount of carbomer 940 and soaked it in water in the refrigerator at 4 °C overnight until it fully swelled. Then, we dropped the triethanolamine with magnetic agitation to adjust the pH value, added an appropriate amount of glycerol to make a gel matrix, an appropriate amount of liposome suspension, an appropriate amount of liposome suspension, and an appropriate amount of water to the gel matrix, and then evenly ground the liposome gel to make P-ACPLG.

### 2.6. Uniform Experimental Design

#### 2.6.1. Design of Comprehensive Evaluation Form

The 2015 edition of the Chinese Pharmacopoeia (Part 4) stipulates that the appearance of the gel should be uniform, delicate, that it can remain gelatinous at room temperature, and that it will not dry out or liquefy. Therefore, the evaluation indexes were determined as the appearance of the gel, its coating ductility, uniformity, discoloration time, and viscosity. The specific evaluation indexes are shown in Table 1, and the molding of the gel was studied. Prior to the test session, the grading team members were given a brief description of the overall grading procedure. The coded gels were provided to the ethics committee of Jilin Agricultural University (232692HJ0102116492, 10 May 2023) for the trained sensory evaluation members (*n* = 10) in random order in centrifuge tubes.

#### 2.6.2. Determination of Entrapment Efficiency

The centrifugal method was used to determine the entrapment efficiency. First, 1.0 mL of polysaccharide liposome gel was precisely measured in a centrifuge tube, placed in a frozen centrifuge, and centrifuged at 10,000 r·min^−1^ for 30 min. After absorbing 0.5 mL of supernatant and adding PBS solution to 100.0 mL, the total polysaccharide content was determined by the phenol-concentrated sulfuric acid method [26].

#### 2.6.3. Uniform Design Experiment

Factors such as the dosage of carbomer 940, liposome, glycerol, and pH value were investigated, and the encapsulation rate and comprehensive score were used as indicators to select six levels (pseudo-level) for each factor. Other factors affecting the quality of the liposomes were tested under fixed conditions and parallel operation methods according to uniform design table U6(6^4^), as shown in Table 2.

### 2.7. Storage Stability

To determine the effect of storage conditions on the leakage rate of the liposome and liposome gel, we transferred 4 mL from each sample into test tubes and stored them at refrigerated temperature (−4 °C), room temperature (25 °C), and frozen temperature (−20 °C) for 28 days. The frozen sample was melted by repeated eddy mixing at room temperature. Then, the change in the sample leakage rate was measured.

### 2.8. In Vitro Drug Release Experiment

In vitro drug release was measured by dialysis. Neutral PBS (pH = 7.4) was selected as the release medium, and 2 mL 1 mg/mL of phosphorylated *Auricularia cornea* var. Li. polysaccharides and phosphorylated *Auricularia cornea* var. Li. polysaccharide liposomes were added into the dialysis bag (3000 KD), respectively. Both ends of the dialysis bag were sealed and placed in a 20 mL release medium at 37 °C ± 0.5 °C to simulate the skin environment. At specific intervals (1, 2, 4, 6, 8, 10, 12, 24, 48, 72, 96 h), 2 mL of the release medium was removed each time into a 10 mL centrifuge tube, and an equal amount of new release medium was added to the beaker. The sample in the centrifuge tube was quantitatively determined by the phenol-concentrated sulfuric acid method (*n* = 3).

### 2.9. Determination of Antioxidant Activity In Vitro

#### 2.9.1. Determination of DPPH Free Radical Scavenging Ability

The samples (ACP, P-ACP, P-ACPL, P-ACPLG) were prepared (0.25 mg/mL, 0.5 mg/mL, 1.0 mg/mL, 2.0 mg/mL, 4.0 mg/mL) at 1 mL with different concentration gradients. We added 1.0 mL of 0.2 mmol/L of DPPH radical ethanol solution (ready to use) and 1.0 mL of the sample solution, shook it well, and placed the prepared solution in room temperature for 30 min away from light. With anhydrous ethanol as the blank control and L-ascorbic acid as the positive control, the absorbance value was measured at 517 nm and the DPPH free radical clearance was calculated. The calculation formula is as follows:

(1) $$\mathrm{Formula}:\;X=(1-\frac{A_{2}-A_{0}}{A_{1}})\times 100\%$$

where *X* represents the DPPH free radical clearance, %; *A*_1_ represents the absorbance value of the blank control (anhydrous ethanol replaces the sample); *A*_2_ represents the absorbance value of the sample test; and *A*_0_ represents the absorbance value of the sample interference test (anhydrous ethanol instead of the DPPH solution).

#### 2.9.2. Determination of Scavenging Ability of Hydroxyl Radical (·OH)

We added 1 mL samples (ACP, P-ACP, P-ACPL, P-ACPLG) of different concentrations (0.25 mg/mL, 0.5 mg/mL, 1.0 mg/mL, 2.0 mg/mL, 4.0 mg/mL) and ferrous sulfate (1.0 mL, 6 mmol/L) into test tubes successively. Salicylic acid (1.0 mL, 6 mmol/L) and 1 mL of hydrogen peroxide (0.03%, *v*:*v*) were shook and mixed. Then, they were placed in a 37 °C water bath for 30 min with anhydrous ethanol as a blank control and L-ascorbic acid as a positive control. The absorbance value was determined at 510 nm.

(2) $$\mathrm{Formula}:X=(1-\frac{A_{2}-A_{0}}{A_{1}})\times 100\%$$

where *X* represents the hydroxyl radical clearance, %; *A*_1_ represents the absorbance value of the blank control (anhydrous ethanol replaces the sample); *A*_2_ represents the absorbance value of the sample test; and *A*_0_ indicates the absorbance value of the sample interference test (anhydrous ethanol instead of hydrogen peroxide solution).

#### 2.9.3. Reducing Power Test

The 1.0 mL sample (ACP, P-ACP, P-ACPL, P-ACPLG) solution and 1.0 mL potassium ferricyanide solution (1%, *w*/*v*) were added to the test tube successively. After full shock and mixing, the reaction was carried out in a water bath at 50 °C for 30 min. After rapid cooling with running water, 1.0 mL of 10% trichloroacetic acid was added. After centrifugation at 6000 r/min for 10 min, 500 μL of supernatant was added to 30 μL of ferric chloride solution (0.1%, *w*/*v*), mixed, and reacted at room temperature for 5 min. Anhydrous ethanol was used as the blank control and L-ascorbic acid was used as the positive control. The absorbance value was determined at 700 nm.

### 2.10. Data Processing

All experiments were measured and repeated three times. Origin 2018 software was used for mapping and DPS7.5 software was used for stepwise regression analysis of experimental data. Significant differences were considered when *p* < 0.05.

## 3. Results and Discussion

### 3.1. Determination of the Phosphate Content of Phosphorylated Auricularia cornea var. Li. Polysaccharides

The molecular structure of polysaccharides can be modified by certain chemical methods that can increase or endow the activity of polysaccharides and reduce their toxic side effects. In this study, the phosphoric acid method was used [27]. The phosphoric acid content in phosphorylated *Auricularia cornea* var. Li. polysaccharide is one of the evaluation indexes of the degree of phosphorylation modification, and the content of phosphoric acid affects the strength of its biological activity [28]. Therefore, the molybdenum blue colorimetric method was used to determine the content of phosphoric acid, with the content of KH_2_PO_4_ as the horizontal coordinate and the absorbance value as the vertical coordinate, and the standard curve was drawn. The fitted linear equation is y = 0.0617x + 0.0473, R^2^ = 0.9979 > 0.99, indicating good linearity and successful establishment of the standard curve. According to the formula calculated in Section 2.3.2, the phosphoric acid content was 15.51%, as measured by the method of Wang [29], and the phosphoric acid content was good and could be used for subsequent preparation.

### 3.2. Determination of Structure Characterization of Liposomes of Phosphorylated Auricularia cornea var. Li. Polysaccharides

As a kind of natural polysaccharide, ACP has the characteristics of high molecular weight. P-ACP is prepared by the phosphorylation of natural polysaccharides, which can change the original structure, and the change in structure will lead to an improvement of physical and chemical properties and the enhancement of biological activity [30]. Liposomes can be used as a drug encapsulation technology, and their composition is similar to that of natural cell membranes to reduce drug toxicity [31]. Liposomes can also be used as a drug reservoir to reduce the drug diffusion rate, achieve a slow release effect, and prolong the drug effect time [32]. Therefore, the preparation of P-ACPL is particularly important.

#### 3.2.1. Determination of Particle Size Distribution, Zeta Potential, and Polydispersion Index of Phosphorylated *Auricularia cornea* var. Li. Polysaccharide Liposomes

**Figure 1:**
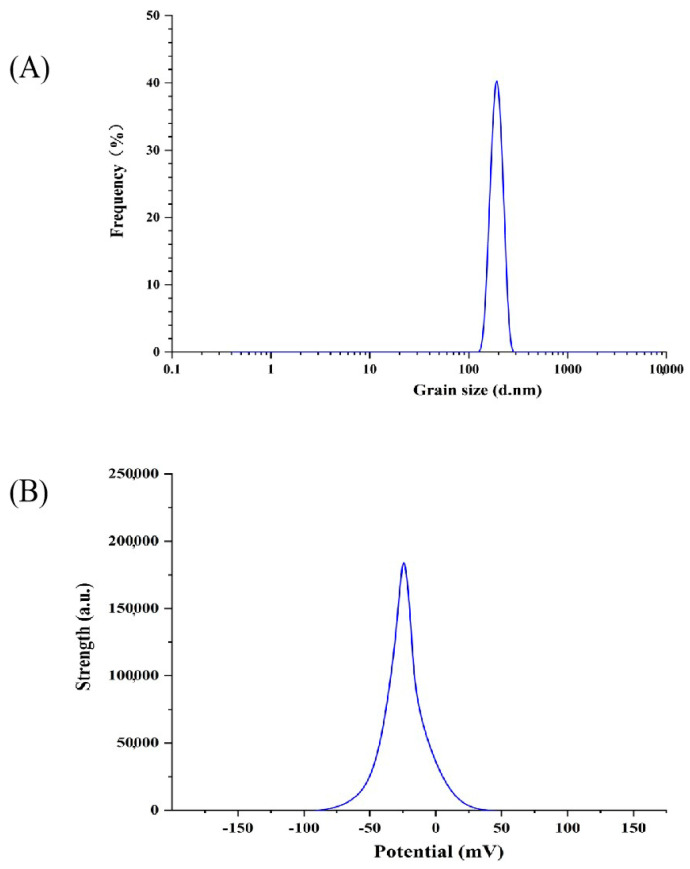
Zeta potential and particle size distribution of P-ACPL (phosphorylated *Auricularia cornea* var. Li. Polysaccharide liposomes): (**A**) particle size distribution, (**B**) potential distribution.

The polydispersion index (PDI) can be used as one of the indicators of the size distribution range of liposomes [33]. The smaller the PDI value, the more concentrated the size distribution range. The diameter of particles is more uniform [34]. As can be seen in Figure 1A, the average particle size of the P-ACPL was 192.2 ± 3.3 nm, the particle size distribution map had no heterogenous peaks, and the polydispersion index (PDI) was 0.134 ± 0.021. The results show that the particle size distribution range of P-ACPL is relatively concentrated and has good uniformity. The potential stability of the liposome system can be further determined by the zeta potential of the particle surface charge [35]. As can be seen in Figure 1B, the zeta potential value of P-ACPL is −30.6 mV. The zeta potential can be used to describe the strength of the interaction between the mutual attraction or repulsion of the plastid particles, and the higher the value, the more stable the liposomes are in the system [36,37]. The average zeta potential of phosphorylated *Auricularia cornea* var. Li. polysaccharide liposomes were −33.4 ± 0.57 mV. The results show that the P-ACPL system has good stability at this potential, and, because the potential is negatively charged, it is an anionic liposome.

#### 3.2.2. Transmission Electron Microscope Observation

**Figure 2:**
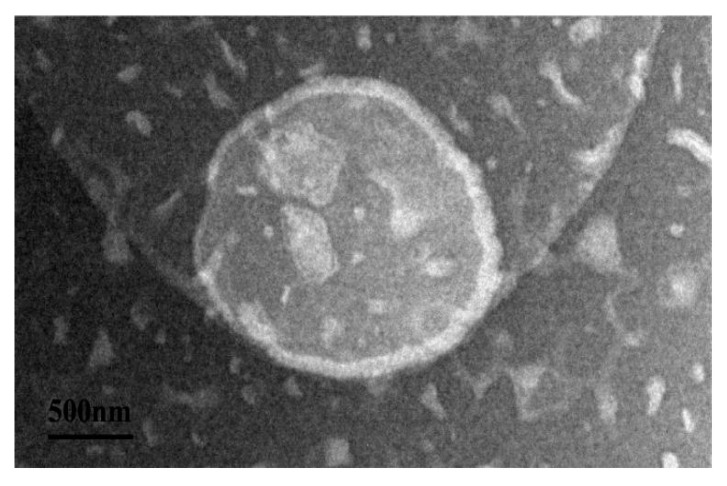
Transmission electron microscopy of P-ACPL (Phosphorylated *Auricularia cornea* var. Li. polysaccharide liposomes) (500 nm).

In order to understand the encapsulation of P-ACPL, the microscopic morphology of P-ACPL was observed by transmission electron microscopy. It can be seen in Figure 2 that the blank liposome (BL) and P-ACPL observed by transmission electron microscopy are spheroidal, uniform in size, round, and non-adherential. Moreover, it can be seen from the comparison between the BL and P-ACPL that the P-ACP was wrapped in the lipid bilayer.

### 3.3. P-ACPLG Uniform Design Optimization Experiment

The experimental results in Table 3 were analyzed by quadratic polynomial stepwise regression with DPS7.5 software. The regression model of each variable (X_1_, X_2_, X_3_, X_4_) and comprehensive score (Y_1_) was analyzed as follows: 0.54 + 0.13 × X_2_ + 2.13 × X_1_^2^ + 0.13 × X_4_^2^ + 18.39 × X_1_ × X_4_. The regression equation was tested and an R^2^ correlation coefficient test value of 0.99 was obtained. There is a close correlation between the comprehensive score and the experimental factors in the regression equation. The significance test value was F = 124999.81, the remaining standard deviation S value was 0.0005, and the significance level was *p* = 0.0021 < 0.01, so the regression equation reached an extremely significant level, indicating that the reliability of the equation was high. The regression model analyzing the respective variables (X_1_, X_2_, X_3_, X_4_) and the comprehensive score (Y_2_) was: 96.16 − 13.32 × X_1_^2^ + 12.78 × X_4_^2^ + 0.33 × X_2_ × X_3_ − 9.16 × X_3_ × X_4_. The regression equation was tested, and an R^2^ correlation coefficient test value of 0.99 was obtained. There is a close correlation between the comprehensive score and the experimental factors in the regression equation. Its significance test value was 54501.23, the residual standard deviation S value was 0.0062, and the significance level was *p* = 0.0032 < 0.01, so the regression equation reached an extremely significant level, indicating that the reliability of the equation was high.

The test results of regression coefficient t of the various experimental factors in the Y_1_ equation are shown in Table 4. It can be seen in Table 4 that the significant levels of X_1_X_4_ in the equation were all less than 0.01, indicating that the interaction between carbomer 940 and liposome had a great impact on the comprehensive evaluation of the gel, reaching a very significant level. The results of the sample observations, fitting values, and fitting errors are shown in Table 4. The data in Table 5 show that the observed values are very close to the fitting values, and the maximum fitting error is −0.0003, which further explains the accuracy of the regression equation. Through the simulation of regression equation Y_1_, the optimum preparation conditions of the gel were obtained as the extraction time of carbomer 940 0.10 g, pH = 5.68, glycerol 0.55 g, and liposome 0.028 g. The highest comprehensive evaluation was 3.06 and the encapsulation rate was 97.65%.

The regression coefficient t-test results of the various experimental factors in the Y_2_ equation are shown in Table 6. It can be seen in Table 6 that the significant levels of X_2_X_3_ and X_3_X_4_ in the equation were both less than 0.01, indicating that the interaction between the pH value and glycerol, as well as between the glycerol and liposome, had a great influence on the gel’s entrapment rate, reaching a very significant level. The results of the sample observations, fitting values, and fitting errors are shown in Table 7. The data in Table 7 show that the observed values are very close to the fitting values, and the maximum fitting error is −0.0049, which further explains the accuracy of the regression equation. Through the simulation of regression equation Y_2_, the preparation conditions of the gel were obtained as the extraction time of carbomer 940 0.11 g, pH = 5.94, glycerol 0.54 g, and liposome 0.027 g. The highest comprehensive evaluation was 3.19 and the encapsulation rate was 98.31%; these are the best process parameters. Verification experiments on P-ACPLG show that the experimental results have good reproducibility, and that the preparation process is stable.

### 3.4. Analysis of Storage Stability of Phosphorylated Auricularia cornea var. Li. Polysaccharide Liposome Gel

**Figure 3:**
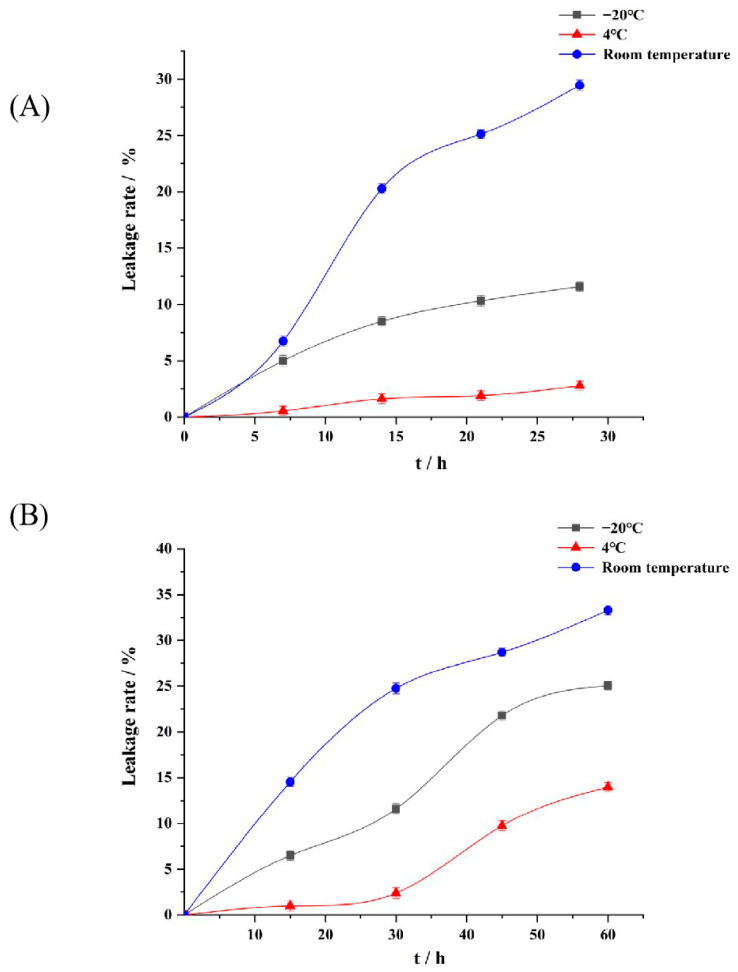
Storage stability of P-ACPLG (Phosphorylated *Auricularia cornea* var. Li. polysaccharide liposome gel) at room temperature (25 °C), refrigeration at 4 °C, and freezing at −20 °C (*p* < 0.05), (**A**) P-ACPL storage stability for 28 days; (**B**) Stability of P-ACPLG storage for 60 days (*n* = 3).

As can be seen in Figure 3A, the appearance of P-ACPL did not change significantly after it was placed at room temperature, 4 °C, and −20 °C for 28 days, and precipitation occurred at room temperature. The packet encapsulation rate of P-ACPL was 85.74%. The encapsulation rates of P-ACPL at −20 °C, 4 °C, and room temperature were 75.80%, 83.36%, and 60.48%, and the leakage rates were 11.59%, 2.78%, and 29.46%, respectively. In summary, P-ACPL is suitable for storage at 4 °C and has the characteristics of small morphological change, low leakage rate, and strong stability. The increase in P-ACP leakage may be due to the increased mobility and oxidation rate of phospholipids at higher or lower temperatures [38].

Due to the accumulation of colloidal particles caused by the long-term storage of liposomes [39], P-ACPLG was prepared by dispersing liposomes into a gel matrix. As shown in Figure 3B, after the P-ACPLG was stored at room temperature, 4 °C, and −20 °C for 60 days, the encapsulation rates of the P-ACPLG at −20 °C, 4 °C, and normal temperature were 65.63%, 75.32%, and 58.41%, and the leakage rates were 25.05%, 13.98%, and 33.29%, respectively. In summary, P-ACPLG is suitable for storage at 4 °C and has the characteristics of small morphological change, low leakage rate, and strong stability.

### 3.5. Determination of Phosphorylated Auricularia cornea var. Li. Polysaccharide Liposome Gel Release In Vitro

**Figure 4:**
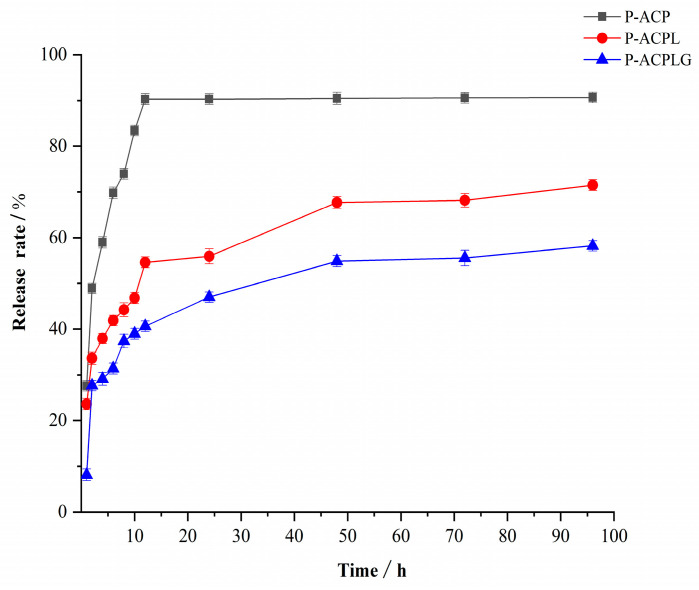
Release curves of P-ACPL (Phosphorylated *Auricularia cornea* var. Li. polysaccharide liposome) and P-ACPLG (Phosphorylated *Auricularia cornea* var. Li. polysaccharide liposome gel) in vitro (*n* = 3, *p* < 0.05).

As can be seen in Figure 4, the in vitro release of P-ACPL and P-ACPLG was investigated, and the results of the comparison with P-ACP show that the release rate of the P-ACP group reached 83.45% at 10 h and was completely released at 12 h. The P-ACPL and P-ACPLG were released rapidly within the first 2 h, with release rates of 35.70% and 27.66%, respectively. From 2 h to 48 h, the P-ACP and P-ACPL had a slow release process and then tended to be smooth. At 48 h, the P-ACPL and P-ACPLG were released completely, and their release rates were 64.94% and 54.87%, respectively. Compared to the P-ACPL, the sustained release effect of the P-ACPLG is more obvious. This is due to the reduced drug release rate of liposome gels, but the release time is longer, and the dosage form is more stable. Because the gel itself hinders the release of drugs [40], the liposome is dispersed in the gel, which improves the stability of the liposome.

### 3.6. Determination of In Vitro Antioxidant Activity

The determination of the scavenging capacity and reducing the capacity of DPPH free radicals and hydroxyl free radicals is an important index to judge the antioxidant capacity [41,42]. In this study, the scavenging ability and reducing ability of DPPH free radicals and hydroxyl free radicals were used as the dependent variables to explore the in vitro antioxidant capacity of the liposome gel before and after emulsification.

#### 3.6.1. Determination of DPPH Free Radical Scavenging Ability

**Figure 5:**
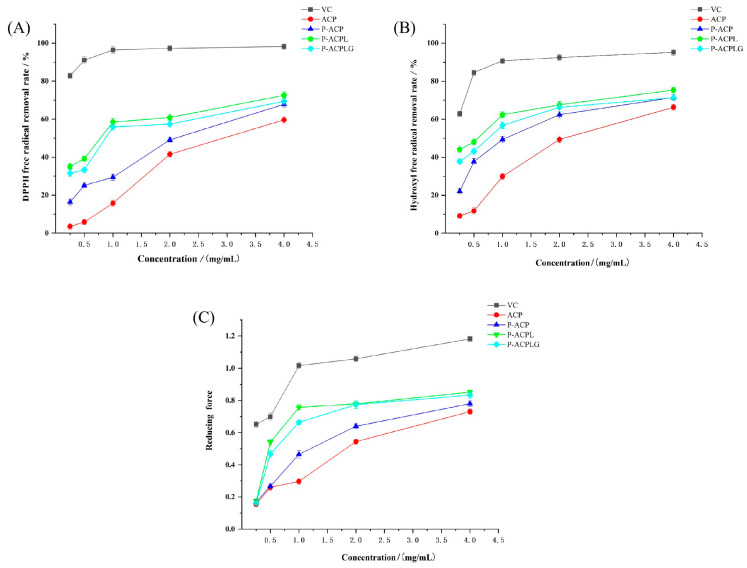
Determination of the antioxidant activity of L-ascorbic acid, ACP (*Auricularia cornea* var. Li. polysaccharide), P-ACP (Phosphorylated *Auricularia cornea* var. Li. polysaccharide), P-ACPL (Phosphorylated *Auricularia cornea* var. Li. polysaccharide liposome), and P-ACPLG (Phosphorylated *Auricularia cornea* var. Li. polysaccharide liposome gel) in vitro, (**A**) determination of DPPH free radical scavenging ability; (**B**) Determination of hydroxyl radical scavenging capacity; (**C**) Determination of reducing power (*n* = 3, *p* < 0.05).

DPPH is a stable free radical centered on nitrogen [43], and it provides a simple and rapid method for the determination of antioxidant activity [44]. The scavenging activity of different concentrations of L-ascorbic acid, ACP, P-ACP, P-ACPL, and P-ACPLG on the DPPH free radicals was studied, and the results are shown in Figure 5A. Within the concentration range of 0.25 mg/mL^−4^ mg/mL, the DPPH scavenging ability of L-ascorbic acid, ACP, P-ACP, P-ACPL, and P-ACPLG increased with the increase in concentration. The DPPH free radical scavenging activity was obviously less than that of L-ascorbic acid, and it was concentration-dependent. At 4 mg/mL, the DPPH free radical scavenging rates of L-ascorbic acid, ACP, P-ACP, P-ACPL, and P-ACPLG were 98.20%, 59.64%, 67.83%, 72.51%, and 69.37%, respectively. The DPPH free radical scavenging ability of P-ACPLG was increased by 3.14% compared to P-ACPL. The DPPH free radical scavenging rate of P-ACPLG was always slightly lower than that of P-ACPL, the IC50 value of P-ACPLG was 1.037, and the IC50 value of P-ACPL was 0.789, which was lower than that of P-ACPL. However, the P-ACPLG still has significant scavenging activity. The reason may be that carbomer 940 can wrap P-ACPL to avoid the oxidation of polysaccharide, resulting in a slight decrease in the DPPH free radical scavenging ability of P-ACPL, but greatly retaining the DPPH free radical scavenging ability of P-ACPL.

#### 3.6.2. Determination of Scavenging Ability of Hydroxyl Radical (OH)

Hydroxyl radicals have high reactivity, so the determination of hydroxyl radical clearance is an important index to reflect the antioxidant activity [45]. As shown in Figure 5B, the hydroxyl radical scavenging ability of L-ascorbic acid, ACP, P-ACP, P-ACPL, and P-ACPLG showed an obvious scavenging effect on hydroxyl free radicals in a concentration-dependent manner within the concentration range of 0.25 mg/mL to 4 mg/mL, but their scavenging ability on hydroxyl free radicals was inferior to that of L-ascorbic acid. When the concentration was 4 mg/mL, the hydroxyl radical scavenging rates of L-ascorbic acid, ACP, P-ACP, P-ACPL, and P-ACPLG were 95.14%, 66.23%, 71.43%, 75.32%, and 71.30%, respectively. The hydroxyl radical scavenging ability of P-ACPLG decreased by 5.20% compared to that of P-ACPLG. The DPPH free radical scavenging rate of P-ACPLG was always better than that of P-ACPL, and the IC50 values of P-ACPLG and P-ACPL were 0.905 and 0.448, which were lower than the IC50 values of P-ACPLG, but P-ACPLG still had significant activity. The reason may be that carbomer 940 plays a coating role on P-ACPL to avoid the oxidation of the polysaccharides, resulting in a slight decrease in the hydroxyl radical scavenging ability but greatly retaining the hydroxyl radical scavenging ability of P-ACPL.

#### 3.6.3. Determination of Total Reducing Power

The determination of the reducing power of natural products is one of the indexes to evaluate the antioxidant activity of substances in vitro [46]. The reducing power of P-ACPLG is shown in Figure 5C. In the concentration range of 0.25 mg/mL^−4^ mg/mL, the higher the absorbance of L-ascorbic acid, ACP, P-ACP, P-ACPL, and P-ACPLG, and the stronger the reducing power is. The reducing capacity increased with the increase in concentration, but the reducing capacity of L-ascorbic acid was significantly higher than that of other samples. At 4.0 mg/mL, the reducing power of L-ascorbic acid, ACP, P-ACP, P-ACPL, and P-ACPLG were 1.182, 0.761, 0.846, 0.852, and 0.803, respectively. The reason may be that carbomer 940 plays a role in wrapping P-ACPL, avoiding the oxidation of polysaccharide, and resulting in a slight reduction in the reducing power of P-ACPL but greatly retaining the reducing power of P-ACPL.

## 4. Conclusions

The preparation and in vitro antioxidant activity of P-ACPLG were studied in this paper. The results show that P-ACPL prepared by the reverse evaporation method is spherical in appearance. The experimental results of the storage stability of P-ACPL show that the storage stability of P-ACPL at 4 °C is higher than that at room temperature and -20 °C. However, due to the accumulation of colloidal particles caused by the long-term storage of liposomes, the P-ACPLG was prepared by dispersing liposomes into a gel matrix. The experimental results of the storage stability of P-ACPLG show that it is suitable for storage at 4 °C, and has the characteristics of a low leakage rate and strong stability. The results of its in vitro antioxidant activity show that with the increase in the P-ACPL concentration, the scavenging rate of DPPH free radicals and hydroxyl free radicals and the reducing ability of P-ACPL increased gradually, and the scavenging rate of P-ACPL was slightly stronger than that of ACP and P-ACP. After emulsifying the P-ACPL into P-ACPLG and coating the gel matrix, the DPPH, hydroxyl radical scavenging rate, and reducing ability of the P-ACPL were unchanged. In future studies, P-ACPLG can be used as a new type of antioxidant. The mechanism of liposome gel on the skin needs further study.

## Figures and Tables

**Table 1:** Comprehensive evaluation table.

Index	Requirement	Scoring Standards	Score
Appearance	Smooth surface, glossy translucent, semi-solid material	The surface is smooth, glossy, translucent, semi-solid	3–4 (Superior)
		The surface has small particles, dull, poor transparency, the appearance color is turbidity and semi-solid state	2–2.9 (medium)
		The surface has particles, dull, poor transparency, and the appearance color is cloudy and liquid	1–1.9 (excellence)
		The surface is rough and granular, dull, opaque, cloudy in color, and liquid	0–0.9 (poor)
Spreadability	Easy to apply, smooth;	Easy to coat, smooth	3–4 (Superior)
		Good coating property	2–2.9 (medium)
		Better coating, rough	1–1.9 (excellence)
		Poor coating, difficult to evenly spread, rough	0–0.9 (poor)
Homogeneity	The whole is uniform and fine, without large clumps	Uniform and fine, no large clumps, slight bubbles	3–4 (Superior)
		More delicate, no clumps, more bubbles	2–2.9 (medium)
		A little rough, there are a few clumps, more bubbles	1–1.9 (excellence)
		Rough, with lots of clumps, with lots of bubbles	0–0.9 (poor)
Color change time	55 °C constant temperature water bath heating, 4 h after the color did not become dark	The color did not become darker after 4 h	3–4 (Superior)
		The color does not become darker after 2 h	2–2.9 (medium)
		The color did not become darker after 1 h	1–1.9 (excellence)
		The color becomes darker after 1 h	0–0.9 (poor)
Stickiness	Moderate viscosity	Moderate viscosity	3–4 (Superior)
		Slightly thick or thin	2–2.9 (medium)
		Thicker or thinner	1–1.9 (excellence)
		Severely caked or too thin	0–0.9 (poor)

**Table 2:** Uniform design table of P-ACPLG (Phosphorylated *Auricularia cornea* var. Li. Polysaccharide liposome gel).

Test Number	A Carbomer 940/g	B ph-Values	C Glycerinum/g	D Lipidosome/g
1	0.3	5.5	3.0	0.05
2	0.6	6.0	2.5	0.2
3	0.2	6.5	2.0	0.8
4	0.5	7.0	1.5	0.025
5	0.1	7.5	1.0	0.1
6	0.4	8.0	0.5	0.4

**Table 3:** Results of uniform design of P-ACPLG (Phosphorylated *Auricularia cornea* var. Li. polysaccharide liposome gel), the entrapment efficiency is measured by three parallel averages for each data.

Test Number	X_1_ Carbomer 940/g	X_2_ pH-Values	X_3_ Glycerinum/g	X_4_ Lipidosome/g	Y_1_ Comprehensive Evaluation	Y_2_ Entrapment Efficiency/%
1	0.3	5.5	3.0	0.05	2.91	85.87
2	0.6	6.0	2.5	0.2	3.02	93.23
3	0.2	6.5	2.0	0.8	2.85	94.84
4	0.5	7.0	1.5	0.025	2.52	82.35
5	0.1	7.5	1.0	0.1	2.33	93.50
6	0.4	8.0	0.5	0.4	2.50	93.68

**Table 4:** Effect of Y1 regression coefficient on comprehensive score.

Regression Term	Partial Correlation	*t*-Value Test	*p* Value
r(y_1_,X_2_)	1.0003	26.8343	0.0258 *
r(y_1_,X_3_^2^)	1.0000	116.1036	0.0057 **
r(y_1_,X_4_^2^)	1.0010	195.3950	0.0035 **
r(y_1_,X_2_X_3_)	1.0795	34.2549	0.0193 *

* Significant (*p* < 0.05). ** Very significant *p* < 0.01.

**Table 5:** Y_1_ Observations, fitting values and fitting error results.

Samples	Observed Value	Fitted Value	Error of Fitting
1	3.0857	2.9897	0.0003
2	3.0030	3.2588	−0.0007
3	2.8699	2.8697	0.0003
4	2.6593	2.4892	0.0009
5	2.5304	2.3309	−0.0009
6	2.5175	2.6697	0.0003

**Table 6:** Effect of Y_2_ regression coefficient on comprehensive score.

Regression Term	Partial Correlation	*t* -Value Test	*p* Value
r(y_2_,X_1_)	−1.0689	83.3821	0.0086 **
r(y_2_,X_1_^2^)	1.0269	74.7576	0.0099 **
r(y_2_,X_1_X_4_)	1.0609	104.1613	0.0070 **
r(y_2_,X_2_X_3_)	−1.0603	28.9916	0.0229 *

* Significant (*p* < 0.05). ** Very significant *p* < 0.01.

**Table 7:** Y_2_ Observations, fitting values and fitting error results.

Samples	Observed Value	Fitted Value	Error of Fitting
1	91.0222	88.5875	0.0312
2	101.7139	93.7731	0.0177
3	103.4704	100.5668	−0.0375
4	95.0261	92.3716	−0.0433
5	95.1830	101.9980	−0.0102
6	99.3945	100.1202	0.0228

## Data Availability

The original contributions presented in the study are included in the article, further inquiries can be directed to the corresponding author.

## Abbreviations

ACP	*Auricularia cornea* var. Li. Polysaccharides
P-ACP	Phosphorylated *Auricularia cornea* var. Li. polysaccharide
P-ACPL	Phosphorylated *Auricularia cornea* var. Li. polysaccharide liposome
P-ACPLG	Phosphorylated *Auricularia cornea* var. Li. polysaccharide liposome gel
PDI	polydispersion index
BL	Blank liposome
